# The Roles of ROS in Cancer Heterogeneity and Therapy

**DOI:** 10.1155/2017/2467940

**Published:** 2017-10-16

**Authors:** Paulo Luiz de Sá Junior, Diana Aparecida Dias Câmara, Allan Saj Porcacchia, Pâmela Maria Moreira Fonseca, Salomão Doria Jorge, Rodrigo Pinheiro Araldi, Adilson Kleber Ferreira

**Affiliations:** ^1^Mogi das Cruzes University (UMC), Villa Lobos Campus, Sao Paulo, SP, Brazil; ^2^Laboratory of Genetics, Butantan Institute, Sao Paulo, SP, Brazil; ^3^Morphology and Genetic Department, University Federal of Sao Paulo, Sao Paulo, SP, Brazil; ^4^University of Sao Paulo, Sao Paulo, SP, Brazil; ^5^Department of Immunology, Laboratory of Tumor Immunology, Institute of Biomedical Science, University of Sao Paulo, Sao Paulo, SP, Brazil

## Abstract

Cancer comprises a group of heterogeneous diseases encompassing high rates of morbidity and mortality. Heterogeneity, which is a hallmark of cancer, is one of the main factors related to resistance to chemotherapeutic agents leading to poor prognosis. Heterogeneity is profoundly affected by increasing levels of ROS. Under low concentrations, ROS may function as signaling molecules favoring tumorigenesis and heterogeneity, while under high ROS concentrations, these species may work as cancer modulators due to their deleterious, genotoxic or even proapoptotic effect on cancer cells. This double-edged sword effect represented by ROS relies on their ability to cause genetic and epigenetic modifications in DNA structure. Antitumor therapeutic approaches may use molecules that prevent the ROS formation precluding carcinogenesis or use chemical agents that promote a sudden increase of ROS causing considerable oxidative stress inside tumor mass. Therefore, herein, we review what ROS are and how they are produced in normal and in cancer cells while providing an argumentative discussion about their role in cancer pathophysiology. We also describe the various sources of ROS in cancer and their role in tumor heterogeneity. Further, we also discuss some therapeutic strategies from the current landscape of cancer heterogeneity, ROS modulation, or ROS production.

## 1. Introduction

Cancer is the second leading cause of death worldwide and accounted for 8.8 million deaths in 2015. Lung, prostate, colorectal, stomach, and liver cancer are the most common types of cancer in men, while breast, colorectal, lung, cervix, and stomach cancer are the most common among women [1]. Cancer comprises a group of more than one hundred malignancies characterized by distinguishing traits named hallmarks of cancer which may be summarized as hyperproliferation, angiogenesis, insensitivity to antigrowth factors, resistance to apoptosis, migration, escape from destruction by the immune system, inflammation, and genome instability [2, 3]. However, hallmarks of cancer are studied as collective characteristics of cancer cells (and the intermingled stroma); individual cells within a given tumor often depicts variability in these traits, hence conferring different patterns of cellularity to tumors [4].

Although the breakthroughs in cancer therapy experienced recently have considerably improved the quality of life of the patients, many of them still succumb mainly due to cancer complications, which in turn is occasioned by late diagnosis, by the inefficiency of treatments, or by the combination of these factors. Curiously, at the beginning of treatment, the majority of tumors exhibit a substantial sensitivity to current traditional therapies, but often these tumors relapse and anticancer drug resistance is established. Resistance to chemotherapy is closely related to tumor heterogeneity, a phenomenon that was long known but underestimated or even neglected ever since, during the management of cancer patients. The existence of distinct subpopulations of cancer cells which differed regarding carcinogenesis, resistance to therapy, and metastatization capacity was first documented by Heppner, Fidler, and their colleagues [5–9] in the 1970s and 1980s. Currently, a myriad of evidence has pointed out the relevant role played by “mosaicism” represented by heterogeneous populations each one constituting a particular niche harboring numerous genetically distinct subclones that differ in many aspects, mainly in their sensitivities and also in their possible therapeutic targets [10]. Intratumor heterogeneity refers to a cellular mosaicism (heterogeneity) occurs within a tumor, while intertumor heterogeneity relates to heterogeneity across several different tumors. In this context, chemotherapy acts not in all cells in a tumor mass, allowing the surveillance of the refractory cells that will be responsible for the high morbidity and mortality related to cancer relapses [4]. However, where did cancer heterogeneity come from? Several hypotheses have been proposed in an attempt to explain the mechanisms involved in the cancer heterogeneity, and all of them are related, at least in part, to the prevalence of cancer stem cell in tumor bulk, asymmetric division presented by cancer cells, variations in the genetic signature, modulation of gene expression, and posttranslational modifications [11].

Reactive oxygen species (ROS) are pleiotropic molecules or free radicals (molecules containing one or more unpaired electrons in the atomic or molecular orbitals) generated by a myriad of complex mechanisms of which the most relevant is the incomplete oxidative phosphorylation that occurs during biomolecule catabolism, especially in the electronic transport chain. Under homeostasis, the cells are protected from the deleterious effects of ROS because they present enzymatic systems responsible for dismantling these highly reactive molecules or even antioxidant substances capable of inactivating excessive ROS and in turn reducing their toxicity. ROS overproduction, failures in the scavenging mechanisms, or even the insufficiency of antioxidants may lead to ROS accumulation culminating in oxidative stress, a state of a cell which is characterized by the oxidation of essential biomolecules [12]. Previously, the orthodox view of ROS considered them as mere by-products of respiration (that leaks from the electron transport chain) which are naturally or pathologically produced (during pro- and antioxidant mechanisms unbalance) and randomly interact with certain cellular targets such as intracellular lipids, proteins, and DNA, leading to the accumulation of damaged (oxidized) and nonfunctional biomolecules [13]. Currently, a myriad of evidences indicates that ROS are important and pleiotropic signaling molecules [13] actuating as a double-edged sword in physiologic (e.g., when hydrogen peroxide is required for cytokine, insulin, growth factor, AP-1, and NF-kB signaling) and pathologic processes such as neurodegenerative diseases, carcinogenesis, and even cancer heterogeneity [14].

Although a broad range of studies are focused on cancer cells per se, the noncancer cell also plays a crucial role in tumor progression. Nonneoplastic cells associated with tumors, such as cancer-associated fibroblasts (CAF), endothelial cells, immune cells, adipocytes, and pericytes, produce and accumulate large amounts of ROS, especially CAF. Some solid tumors, in particular pancreatic, prostate, and breast cancer, depict numerous CAFs in contrast to a scarce number of these cells usually found in renal and brain cancers [15]. However, all these distinct cells (cancerous or not) associated to peritumoral neoangiogenesis constitute the heterogenic and desmoplastic tissue frequently found in several solid tumors and related to poor prognosis. Desmoplasia creates an auspicious microenvironment for tumor proliferation by originating a hypoxic and acidic niche as well as generating a mechanical stretch that is responsible for converting fibroblast to myofibroblast that also contributes to cancer heterogeneity and drug resistance [16]. Therefore, this altered microenvironment is maintained by hypoxic and acidic microenvironment associated with an oxidative stress fueled by a high level of mitochondrial ROS production [17].

Therefore, herein, we review what are ROS and how they are produced in normal and in cancer cells while providing an argumentative discussion about their role in cancer pathophysiology. We also describe the various sources of ROS in cancer cells and what is known about their role in tumor heterogeneity. We also propose therapeutic strategies that can preclude the tumor progression by modifying the microenvironment and modulating ROS production as well as promoting an improvement of therapeutic agents and, thus, the efficacy of cancer treatment.

## 2. The ROS Chemistry

ROS are molecules that may be radicals such as superoxide (O_2_^•−^) and hydroxyl radical (^•^OH) or nonradicals such as hydrogen peroxide (H_2_O_2_) and singlet oxygen (^1^O_2_) derived from oxygen (O_2_) that can readily oxidize other molecules including lipids, amino acids, proteins, and nucleic acids [18]. The electronic configuration of the oxygen diatom is [2He^4^]2s^4^2p^8^ with the first ten electrons placed into the *σ*, *σ∗*, and *π* orbitals and two unpaired electrons each located in a different *π∗* antibonding orbital. Removal of an electron from O_2_ results in a superoxide cation radical (O_2_^•+^). In contrast, if a single electron is added, the product is the superoxide anion radical (O_2_^•−^) [14]. The anionic nature of O_2_^•−^ restricts both its diffusibility throughout the cell and its reactivity toward electron-rich substrates. A variety of intracellular ROS are derived from superoxide (O_2_^•−^), which is dismutaded (converted) to hydrogen peroxide (H_2_O_2_) by superoxide dismutases (SODs) [19]. In contrast to superoxide, hydrogen peroxide is highly diffusible throughout the cells and reacts with low pKa thiol groups or proteins containing transition metals (e.g., [Fe–S] clusters). In fact, due to electrostatic attraction, [Fe–S] clusters are the main cellular target of (O_2_^•−^)-mediated toxicity. The reduction of H_2_O_2_, catalyzed by the transition metal, produces a high reactive hydroxyl radical (OH^•^) [20], for which there is no antioxidant protection system. Thus, O_2_^•−^/H_2_O_2_ overproduction leads to a whole series of radical chain reactions involving damage of important biomolecules promoting oxidative stress.

Formation of ROS is strongly related to the emergence of several human pathologic conditions such as atherosclerosis, neurodegenerative diseases, and aging as well as certain types of human cancers including lung, breast, and colon. ROS are generated in organisms by *γ*, X, and UV radiation, biotransformation of dietary chemicals, some diet components, for example, transient metal ions and inflammatory reactions during normal cellular metabolism. The resulting disturbance of the prooxidant/antioxidant balance leads to a condition of oxidative stress, with subsequent oxidation of cellular components, activation of cytoplasmic/nuclear signal transduction pathways, modulation of gene and protein expression, and alteration of activities of DNA and RNA polymerases [18].

## 3. Regulation of ROS Production

ROS can appear from numerous intracellular sources. Among them, the most important are mitochondria, NADPH oxidases, and other enzymes. In most cell types, mitochondria are thought to be the main contributor to intracellular ROS production [13]. More specifically, inside the mitochondria, the electron transport chain complexes I and III, which pump protons out of the inner mitochondrial membrane, are the sites responsible for the major superoxide production [21].

Oxidative phosphorylation in mitochondria involves four electron-transporting complexes, besides a H^+^-translocating ATP synthetic complex. The complexes I (NADH-ubiquinone oxidoreductase) and III (ubiquinol-cytochrome *c* oxidoreductase), as said above, were shown to be responsible for much of the superoxide production [22–24]. Mammalian complex I is an assembly of proteins composed of 34 subunits encoded by the nuclear DNA and seven subunits encoded by the mitochondrial DNA (mtDNA). A series of electron carriers that are evolutionarily conserved conduct electrons derived from the initial oxidation of NADH through a series of iron-sulfur centers to eventually reduce ubiquinone by a mechanism that pumps protons from the mitochondrial matrix to escape to the outside of the mitochondrial membrane. This complex generates semiquinones that have been identified as the probable electron donors for transforming O_2_ into O_2_^−^, although the mechanisms are not entirely known yet [22, 25]. Nevertheless, there are two possible points where oxygen could access electrons at the end of cofactor chain: the flavin moiety and the quinine-binding site.

Complex III, in turn, reduces equivalents, generated in complexes I and II and contained in ubiquinol, and transfers them through reactions with cytochrome *b*, the Rieske iron-sulphur protein, and cytochrome *c*_1_ to the final electron acceptor cytochrome *c* [26]. During the mechanism operation of complex III, two species of semiquinones are also generated. The Q-cycle mechanism proposed for the functioning of the ubiquinol cytochrome *c* reductase starts with ubiquinol donating one electron to the Rieske iron-sulphur protein, producing a semiquinone in proximity to the outer face of the inner membrane, which then reduces the first cytochrome *b* heme (*b_1_*). The second cytochrome *b* heme (*b*_H_) located closer to the matrix side of the membrane accepts one electron from the first heme and reduces ubiquinone to form ubisemiquinone and, subsequently, with the passage of another to form ubiquinol [27].

As the structure and the functional mechanisms of complex I are still unclear, a proper manner to study complex I is to inhibit it. This complex is inhibited by more than 60 different family compounds [28], and the rotenone is the most commonly used to inhibit ROS formation during reverse electron transfer and to induce it during forwarding electron transfer [29, 30]. The superoxide production is higher during the reverse electron transfer from succinate to NAD^+^, while the forward electron transfer is lower [31–34]. Complex I inhibiting method also showed that an iron-sulfur cluster could be the site of electron leak (in which oxygen ends up capturing an electron, forming superoxides [29, 30, 35]. Structural modifications in complex I play a crucial role in ROS production mechanism [26]. Mutations in mtDNA, which are constitutive or caused by mtDNA damage, can lead to these modifications, resulting in a variety of pathogenesis and cell aging [36, 37]. A consequence triggered by a domain alteration in the respiratory complexes is the decrease of electron transfer activity, increasing ROS production, and thus establishing a vicious cycle of oxidative stress and energetic decline [38, 39]. This dysregulation of mitochondrial metabolism is considered to be one of the roots of age-related degenerative diseases [37] and cancer.

Cancer cells accumulate alterations in mitochondrial genome, dysregulations in mitochondrial respiration, and increased ROS production, feeding back oxidative injuries and maybe increasing mtDNA mutation rate [40]. Interestingly, complex I genes usually present higher quantity of mutations than the others in various tumors [41]. Depending on the type of mutation, it can be acquired before or after oncogenesis. So, in cancers, would the metabolic dysregulation be the cause the oncogenesis or is it an effect of it? This question remains on debate.

Another problem of mitochondrial ROS accumulation is disruption of the aggregation of complexes I and III instigated by lipid peroxidation. Progressive peroxidation of mitochondrial phospholipids can induce pathophysiology in the respiratory chain [42], especially a decreased activity of complexes I and III [43, 44]. Taking into account that cells differ in morphology, the quantities of vesicles, reticles, organelles, and mitochondria, density, size, and cytoskeleton arrangement, and their susceptibility to ROS damage may also vary according to this. Mitochondria with a thin membrane may be, for example, more susceptible to ROS effects than one with a thicker membrane. As we can see, various factors are proposed to influence ROS generation in mitochondria [21, 45]. However, knowledge about the in vivo regulation of mitochondrial oxidant production still has many blank spaces to be filled.

NADPH oxidase (NOX), another important source of ROS production, is a multisubunit enzyme that catalyzes ∙O_2_^−^ production by the reduction of O_2_ using NADPH as the electron donor. The classical NADPH oxidase found in neutrophils is composed of five subunits: p47phox, p67phox, p40phox, p22phox, and gp91phox (catalytic subunit, also known as NADPH oxidase isoform 2 (NOX2)) [46, 47]. In unstimulated cells, p47phox, p67phox, and p40phox are found in the cytosol, whereas p22phox and gp91phox are in the cell membrane, organized as a heterodimeric flavoprotein, cytochrome b558. In a stimulated cell, however, p47phox is phosphorylated and the cytosolic subunits form a complex that translocates to the cell membrane and associates with cytochrome b558 to assemble the active oxidase, which transfers an electron from the substrate to O_2_, forming ∙O_2_^−^ [48].

Although NOX were originally considered as enzymes present only in phagocytic cells involved in host defense and innate immunity, more recent evidences indicate that there is a whole family of NADPH oxidases, based on the discovery of gp91phox homologs [49, 50]. These newly discovered homologs, expressed in various tissues and involved in many biological functions, are now known as the NOX family of NADPH oxidases [51].

ROS produced by NOX are considered normal and necessary. They have two major roles in this process. The first one is microbial killing, and superoxide generated by NOX2 is required for the respiratory burst that occurs in phagocytes. The second role is associated with regulation of cell signaling. Even so, ROS produced by NOX are capable of specifically and reversibly reacting with proteins, altering their activity, localization, and also half-life [52].

## 4. ROS and Cancer Heterogeneity

Implications of ROS in tumor heterogeneity still lack extensive studies. While certain cancer types or cell subpopulations benefit from ROS-based therapies, oxidative stress potentially instigates unbecoming effects on other cells or subpopulations. Both healthy and cancer cells may have increased genetic instability and mutations possibly caused by free ROS inside the cell or even in the cell niche. The dynamic sequence of events and constant pressure for cell readjustments eventually promote the evolution of resilient, drug-resistant cells. It is questionable if mitochondrial ROS-mediated mechanisms are the unique contributor to cancer drug resistance, but its roles and modulation of metabolic events may be central to the process and results. Cancer cells usually have some alternative survival mechanisms, and the mitochondrial dysfunction and genetic alterations may facilitate some of these cell survival advantages, even in the presence of drug targeting [53].

ROS also impact cancer stem cells. Just as only a small subpopulation of cancer cells resist to therapies, a small portion of cancer stem cells determine cell differentiation. Considering ROS as a mutagenic agent, it can either block self-renewal or stimulate stem cell differentiation [54], which may, consequently, increase tumor heterogeneity. Interconnected or parallel ROS signaling networks may also influence cancer stem cells and thus cancer drug-resistant cells. Such signaling via ROS occurs for example in FoxO, a family of the Forkhead transcription factors, which is strictly regulated by PI3K/Akt. The phosphorylation of FoxO transcription factors 3 (FoxO3) by Akt leads to the FoxO3's association with 14-3-3 proteins and the FoxO3's retention in the cytoplasm. The sequestration of FoxO3 in the cytoplasm decreases the transcription activity of FoxO3-targeted genes such as SOD_2_ and catalase, culminating in the elevation of ROS [55].

Therefore, the ability to coordinately or independently modulate a repertoire of ROS signaling networks that conflict with cellular functions and processes is critical to the regulation of ROS-mediated drug resistance [53].

## 5. Cancer Stem Cell and ROS

The raising of different intratumor subpopulations is attributed at least in part to a subset of stem cell-like populations named cancer stem cells (CSC). Solé et al. suggest that these CSC are genetically unstable and that this instability is derivate from the loss of DNA repair mechanisms and cell-cycle checkpoints [56]. A growing amount of evidences suggest that CSC is closely related to the emergence of cancer heterogeneity.

The CSC hypothesis suggests that a minor subpopulation with stemness properties is responsible for the tumor growth by giving rise to different subclones that are frequently associated to intratumor heterogeneity [57]. Of note, another theory to justify the cancer heterogeneity, known as the clonal evolution model, asserts that a malignant cell accumulates various hereditary changes over the time that implicates in advantages or disadvantages to the cell, which is hence subjected to natural selection. The tumor implementations take place after the accumulation of several mutations in a single cell. However, these hypotheses are not mutually exclusive being complementary of each other [58].

Currently, there is a consensus that CSCs generate cancer heterogeneity via their selective ability to maintain tumor growth through the generation of new copies of themselves before differentiation while originating other differentiated high cycling cell [59, 60]. On the other hand, the stochastic model argues that all malignant cells have the capability to generate the intratumor heterogeneity constituting a hallmark of several cancers and are believed to be induced by genetic and nongenetic mechanisms [61]. The genetic mechanism of cancer heterogeneity comprises direct DNA alterations such as successive mutations or base deletions. These alterations can be also interleaved with ROS which have been increasingly implicated in the physiological regulation of crucial developmental processes, biological processes, from gene expression and protein translation to protein-protein interactions, and so forth. Also, deep DNA injuries are inconsistent with cell viability and frequently are associated with apoptosis, contributing to eliminate genetically abnormal cells and maintaining homeostasis [62]. Thus, although mutations can lead to carcinogenesis, other mechanisms are involved in cancer heterogeneity. Many pieces of evidences have pointed that cancer cell heterogeneity is epigenetically modulated, for instance by the microenvironment. Hence, the expression of proteins related to proliferation, migration and metastasis, apoptosis resistance, and stemness would altogether be controlled by epigenetic events such as DNA methylation, histone modification, and polycomb, miRNA, and chromatin remodeling which in turn are tightly influenced by the cell niche favoring the emergence of heterogeneity [63].

CSCs' influence counterpoises the radiation-induced ROS production by increased expression of free radical scavengers, as reported for mouse and human breast CSCs [64]. In opposition to the high proliferative cancer cells, CSCs display very low levels of ROS, mainly due to increased activity of the antioxidant machinery and aerobic glycolysis. Leukemia stem cells (LSCs) are highly vulnerable to increases in ROS levels [64, 65]. This ability might selectively protect CSCs from ROS-mediated DNA damage and hence explain their resistance to irradiation treatments. Importantly, in vitro studies of drug-sensitive tumor cell lines suggest that cancer cells might transiently and reversibly acquire drug resistance, indicating that drug resistance might not always be a stable trait [66].

Given that ROS may influence a vast array of biological processes, and that we are limited in our knowledge of which species of ROS are implicated in any given physiological setting, it seems an immense challenge to explore how ROS metabolism can be manipulated to generate stem cells and influence stem cell fate [67]. Understated differences between normal cells and CSCs in their sensitivity to ROS can be exploited to target CSCs in therapy.

## 6. The Roles of ROS in Cancer Proliferation

A heterogenic cancer cell population may be developed through stress selection such as hypoxia, lack of nutrients, chronic inflammation, and immune system activation. Tumors with a high degree of heterogeneity frequently demonstrate a high resistance to chemotherapy due, at least in part, to the large number of CSCs [68, 69]. Different molecular targets have been studied in order to identify which therapy would be specific to resistant CSC, including oxidative stress therapy and chronic inflammation, therapies to reverse the quiescent state presented by CSC and therapies aiming at DNA damage and efficient cell export of cytotoxic agents. The CSCs, similar to normal stem cells, are quiescent, slow-cycling cells with the lower level of intracellular ROS when compared to typical high proliferative cancer cells [64, 70]. It is known that low levels of ROS are critical for the stemness of SCs and HSCs [71].

The biological effects of ROS and the mechanisms regulating its level have been studied in cancer cells as a whole, but little is known about these issues specifically in the CSC population. Recent studies have associated ROS, c-Myc, and *β*-catenin-dependent Wnt pathway, which regulate c-Myc, as a model for tumor proliferation.

Wnt signaling is important in embryo development and also controls homeostatic self-renewal in adult tissues [72]. Radioresistant breast cancer cells showed CSC-like properties and elevation of *β*-catenin. NS398, a cyclooxygenase 2 inhibitor, enhanced the radiosensitivity of these cells, which may be partially be explained by the downregulating the expression of *β*-catenin [73].

In cancer cells, ROS are principally generated by a high rate metabolism in mitochondria, endoplasmic reticulum, and cell membranes [74]. The metabolic phenotypes observed in tumor cells are different from the normal tissue. Unlike nontumor cells, most of the energetic supply of cancer cells occurs through increasing rate of glycolysis followed by oxidation of pyruvate in mitochondria, a phenomenon known as Warburg effect [75, 76]. The glycolysis replaces at least part of the oxidative phosphorylation for generation of ATP in cancer cells. This metabolic switch is essential for the cancer cells to adapt to hypoxic conditions with less mitochondrial defects and ROS production [77]. ROS are involved in each stage of cancer development, including initiation, promotion, and progression [78]. The increase in intracellular ROS in cancer cells may involve a diversity of mechanisms. The intrinsic mechanism of increasing intracellular ROS may result from the activation of oncogenes, inactivation of tumor suppressor genes, high metabolism, and mitochondrial dysfunction [78]. It is common in cancers for metabolism to be very active under the drive of oncogenic signals, for example, constitutively active mutant Ras, Bcr-Abl, and c-Myc [79, 80]. The activation of c-Myc could increase ROS without the induction of apoptosis, while the treatment with antioxidant NAC decreased the number of c-Myc-induced hMre11 signals and improved cell survival after c-Myc activation. c-Myc is a transcriptional factor related to cell proliferation and overexpression of this factor has also been found in cervical carcinomas, leukemias, lymphomas, colon, and testicular cancer [55, 81].

During the process of tumor metastasis, which is often enabled by epithelial-mesenchymal transition (EMT) [82], disseminated cancer cells would seem to require a self-renewal capability, similar to that exhibited by stem cells, in order to spawn macroscopic metastases. This raises the possibility that the EMT process, which enables cancer cell dissemination, may also impart a self-renewal capability to disseminating cancer cells. Indeed, the metastatic process is at least superficially similar to the processes that occur during tissue repair and regeneration and enable adult stem cells to abandon the tissue reservoirs, such as the bone marrow, enter and survive in the circulation, and exit into secondary tissue sites, where they proliferate, differentiate, and participate in tissue reconstruction [83, 84]. Together, these various lines of evidence suggested a possible link between less differentiated stem cells and the mesenchymal-appearing cells generated by EMTs.

Mani et al. and Morel et al., established a crucial link between passage through EMT and the acquisition of molecular and functional properties of stem cells [85, 86].

**Figure 1:**
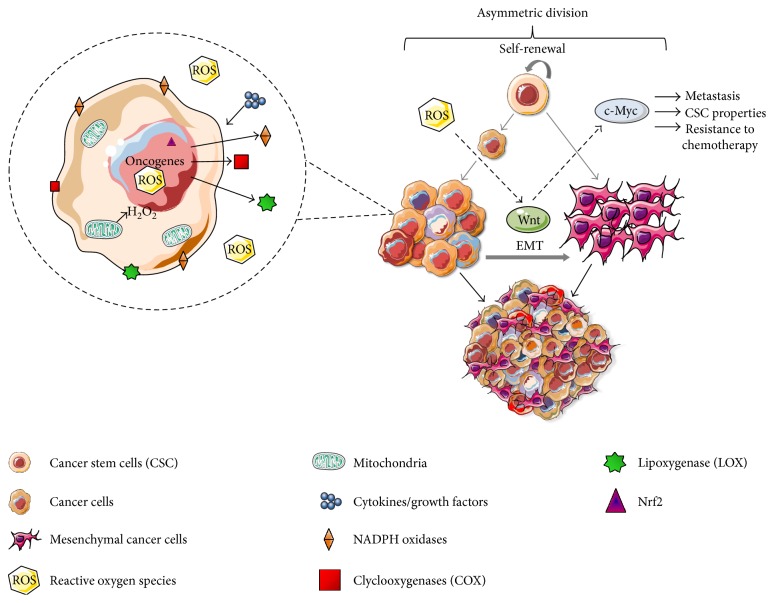
Quiescent and/or self-renewing stem cells display low levels of ROS due to their basal metabolism associated to an efficient antioxidant machinery. ROS can result from increased metabolism associated with dysfunctional mitochondria, oncogene activation, or cytokine/growth factor signaling that triggers ROS-producing enzymes: NADPH oxidases, cycloxygenases (COX), and lipoxygenases (LOX). ROS-induced Wnt/*β*-catenin signal in cancer stem-like cells. The exposure to oxidative stress activates Wnt pathway and upregulates c-Myc. In CSCs, c-Myc expression level varies from cell-to-cell contributing to cancer heterogeneity. Wnt activate downstream signaling molecules that promote the stabilization and accumulation of the *β*-catenin in the nucleus and leading to EMT, a hallmark of cancer.

In summary, ROS can induce Wnt signal, specifically Wnt/*β*-catenin pathway. Wnt activates downstream signaling molecules that stabilize and promote accumulation of the *β*-catenin into the nucleus [87, 88]. Many Wnt ligands have been reported to promote EMT and CSC activities in various types of cancers [89, 90]. c-Myc is regulated by Wnt/*β*-catenin pathway, and consequently, it can attribute greater metastatic potential, CSC properties, and resistance to chemotherapy (Figure 1()).

By itself, a mutagenic agent, ROS possess the ability to block self-renewal or stimulate differentiation of stem cells [54]. Interconnected ROS signaling pathways can influence cancer stem cells and cancer resistant cells. Thus, the ability to coordinate or independently modulate several ROS signaling networks that interfere with cellular functions and processes is critical for the regulation of ROS-mediated drug resistance.

## 7. Oxidative Stress Inducing Genomic Instability and Cancer

Genetic or genomic instability comprises a phenomenon characterized by the high frequency of mutations that occurred within the genome of a particular group of cells, especially neoplastic cells, which has been called “facilitating characteristics” that contributes to set up the hallmarks of cancer while culminating on cancer heterogeneity [91]. The thousands of mutations that characterize the process favor the acquisition or elimination of DNA fragments leading to structural alterations of chromosomes. Especially in cancer, variations in the chromosomal number are a frequent occurrence as well. Both structural changes and variations in the number of chromosomes are common occurrences in many if not all types of cancers [92].

High ROS production and its subsequent accumulation in cells or tissues may favor the interaction of these molecules with DNA components, producing bases modification, inducing inter- and intrastrand bindings or promoting DNA-protein crosslinks leading carcinogenesis [93]. Several examples of ROS-interacting modifications in biomolecules are now recognized, for instance, (i) hydroxyl radicals may react with pyrimidines and/or purines as well as chromatin proteins (ii) or may interact with DNA, causing single- and/or double-strand breaks resulting in base modifications and genomic instability, respectively, all of which can cause alterations in gene expression [94]. The leading oxidative DNA damage products include those of 8-oxo-7,8-dihydroadenine (8-oxoAde), 8-oxo-7,8-dihydroguanine (8-oxoGua), 8-oxo-7,8-dihydro-2-deoxyguanosine (8-oxodG), and 5,6-dihydroxy-5,6-dihydrothymine as well as the ring-open lesions of 4,6-diamino-5-formamido-pyrimidine and 2,6-diamino-4-hydroxy-5-formamido-pyrimidine [18, 94]. The 8-oxoGua constitutes one of the most common products of DNA lesions caused by ROS, because guanine is the most susceptible to oxidation than other nucleobases [95] and strongly implicated in all stages of carcinogenesis. Tissues devoid of efficient machinery for ROS removal are particularly vulnerable to develop mutagenesis and carcinogenesis [96]. Exogenous ROS generators such as smoking or high alcoholic consumption constitute important risk factors since they increase by up to 50% the rate of formation of 8-oxoGua adducts [97]. Furthermore, high rates of 8-oxoGua are commonly observed in several inflammatory pathologies, aging, accelerating telomere shortening, and even in neoplastic tissue in vitro and also in lung, breast, or prostate cancer patients when compared to healthy individuals [95]. Unrepaired 8-oxoGua is potentially one of the most mutagenic lesions among oxidatively modified DNA bases, due to its pairing with A which will cause a GC **→** TA mutation [97]. These findings are suggestive that 8-oxoGua constitutes a prominent candidate to serve as a reliable biomarker of ROS-induced mutagenesis and tumorigenesis or even in theranostics [18].

In addition to the discussion above, high levels of ROS may still lead to 8-oxoGua accumulation in telomeres, mainly because in humans these regions are typically constituted by 10–15 kb multiple repeated sequences composed by in-tandem (TTAGGG)_n_ hexanucleotide repeats [98]. The presence of 8-oxoGua in telomeres inhibits telomerase activity and decreases the binding of telomeric proteins to the telomere sequence, leading to the disruption of telomere length, precluding the maintenance of chromosomal-end capping with several consequences to a vast variety of biological process including ageing, cell death, carcinogenesis, and chromosome instability [99, 100]. Further, telomeres are less efficiently repaired than the other portions of the genome [100]. It has been previously demonstrated that chromosome instability occasioned by telomere dysfunction implies the development of nuclear anomalies such as micronuclei (MN), nucleoplasmic bridges (NPBs), and nuclear buds (NBUDs) that constitute biomarker genotoxicity [99, 101].

## 8. Anticancer Therapy

Heterogeneity contributes negatively to anticancer therapies since multicellularity may favor the emergence of subclones or subpopulations of cells that are refractory to both traditional or target therapies and even to radiotherapy. Chemical reactivity distinguishes ROS from other signaling molecules conferring peculiar characteristics which will later be discussed. Low levels of ROS actively participate in the complex mechanisms of control of cell proliferation and differentiation, while excessive amounts may lead to important cell damage or even to apoptosis. However, a cancerous cell tolerates high levels of endogenous oxidative stress, both in culture and in vivo when compared with their regular counterparts, as a result of an aberrant regulation of redox homeostasis and stress adaptation [102]. The previous study demonstrated that preincubation of normal epithelial cells to low but continuous levels of exogenous oxidants confers cellular resistance to subsequent oxidative stress even at higher concentrations, showing that cancer cells may adapt to survive to oxidative microenvironments [102]. Since cancer cells actively produce and accumulate large amounts of ROS, without suffering the acute and deleterious effects of oxidation, we argue that intrinsic oxidative stress observed in several tumors may exert selective pressure to enrich the tumor bulk with a population of cells that are capable of stress adaptation [102]. Thus, those cells capable of resisting oxidative moiety tensions may originate ROS-resistant daughter cells, and the proliferation of these high ROS-resistant cells may also contribute to cancer heterogeneity.

Previously, Kong and colleagues proposed the “threshold concept” in an attempt to discriminate normal from neoplastic cells based on their differential ability in maintaining homeostasis. The *threshold concept* argues that the cells respond progressively to increasing concentrations of ROS, oscillating from an adaptive proliferation, passing through the equilibrium state, and finally, after the ROS level surpasses certain limits, the cells are eliminated by apoptosis [103]. In normal cells, the mechanisms underlying ROS scavenging, such as enzyme-based or direct antioxidant molecules, constitute an efficient protection system against malignant transformation. In these cells, a sudden ROS rise, referred as “over boost,” may trigger apoptosis, eliminating this cell [103, 104]. Those cells that survive to oxidative insult are presumably capable of acquiring adaptive mechanisms to counteract the potential toxic effects of elevated ROS and to promote cell survival pathway, and these cells usually constitute neoplastic cells.

Based on the above exposition, what is the most effective therapeutic approach to be used in the management of cancer? Prooxidative or antioxidative therapies? The answer for this issue may be much more complicated than it appears to be.

Chemotherapy and radiotherapy are a primordial treatment for several cancers and may be associated or not to cytoreductive resection to eliminate all tumor cells. While the ionizing radiation used in radiotherapy induces a primarily and direct DNA damage, most of the mechanisms underlying chemotherapeutic agents involve ROS production, and consequently promote oxidative damage [105]. Platinum-based antineoplastics such as cisplatin, carboplatin, oxaliplatin, and several alkylating agents induce ROS production [106]. Although these agents are proven to be quite useful in tumor remission in initial chemotherapy, these drugs tend to lose their effectiveness as soon as tumors acquire resistance. Therefore, we propose here that in spite of the increase in ROS production caused by a wide range of chemotherapeutics, only part of cancerous cells are eliminated; the reminiscent cells can repopulate the tumor and contribute to the emergence of a new heterogenic and drug-resistant tumor.

Nuclear factor E2-related factor 2 (Nrf2), a transcription factor that regulates multiple antioxidant and detoxifying enzymes, is primarily involved in adaption to various cellular threatening situations including ROS-mediated stresses [107]. Adaptive stress responses occurring in certain tumor cells may include the activation of redox-sensitive Nrf2, promoting an increase in the expression of ROS-scavenging enzymes, increasing the levels of prosurvival factors and inhibiting cell death factors. Notwithstanding, the knockdown of Nrf2 in endometrial serous carcinoma, a heterogeneous and highly resistant type of cancer, sensitized Nrf2-overexpressing cells to chemotherapy [108]. These findings suggest that therapy aiming at Nrf2 inhibition may preclude cell proliferation while sensitizing the neoplastic cells to apoptosis. Therefore, therapeutic regimens based on the Nrf2 inhibition can become the frontline in cancer management, especially for tumors with a high level of expression of Nrf2 [108].

Epigenetics comprise stable heritable traits of chromosomes without alteration of the DNA sequence by itself [97]. A growing amount of evidences has indicated that in addition to genetic aberrations, epigenetic modifications directly contribute to cancer heterogeneity and drug resistance, and ROS plays a critical role in the mechanisms underlying epigenetic regulation. Epigenetics disorders have been identified in either cell culture or even in vivo especially in tumors with a high degree of heterogeneity. In fact, it constitutes a novel paradigm in cancer biology which posits that tumors are composed of different cell populations, named cancer cells (CS) and cancer stem cells (CSC), besides the noncancerous cells, frequently found in the tumor bulk as previously mentioned [109]. Although other epigenetic changes may also occur, global hypomethylation, particularly at centrometric repeats, and hypermethylation, often occurring in CpG islands, are frequently present in cancer [110, 111]. In this scenario, the use of drugs that interfere with mechanisms of methylation control, especially demethylating agents, may have an important contribution to anticancer therapy. Two classes of demethylating agents are currently available: nucleoside DNMT inhibitors such as 5-azacytidine (azacitidine) and its derivate 5-aza-2′-deoxycytidine (decitabine (DAC)) and nonnucleoside DNMT inhibitors such as hydralazine, a vasodilator used as an antihypertensive agent, procaine, a local anesthetic, epigallocatechin-3-gallate (EGCG), the main polyphenol constituent of green tea, and MG98, a second generation of DNMT. Indeed, the use of these agents for cancer treatment is not a brand new idea. The first demethylating agent, 5-azacythidine (4-amino-1-*β*-D-ribofurnosyl-1,3,5-triazin-2-one), a nucleoside pyrimidine-derivate analog, was first synthesized about 40 years ago in Prague, Czech Republic, and has since demonstrated remarkable biological effects including modulation of cell differentiation and cytostatic activity in various types of cancer [112]. Therefore, although these agents have been known for a long time, only two inhibitors of DNA methyltransferases, 5-azacytidine (Vidaza) and 5-aza-2-deoxycytidine (decitabine), a cytidine analog, with better specificity, remarkable inhibition of DNA methylation, and less toxic effects than 5-azacytidine, have already been approved by the FDA as effective drugs for treatment of myelodysplastic syndromes [12, 113]. Another cytidine-analog nucleoside, Zebularine have been widely investigated basically because it is more stable than 5-azacytidine and decitabine [12]. Among the nonnucleoside DNMT agents, EGCG stands out as a prominent candidate for cancer therapy due to their ability to bind and block the active site of DNMT1. Interestingly, the degradation of EGCG generates considerable amounts of hydrogen peroxide that may contribute to cell death [114].

## 9. Concluding Remarks

Accumulative evidences have been proven that ROS are not merely by-products of respiration. In addition to acting in the signaling process, these molecules play a crucial role during physiological and pathological events including aging, neurodegenerative diseases, and cancer. In recent years, the canonical role of ROS in genetic and genomic instability has been extensively studied, but these studies do not respond to certain aspects of the dynamics of cancer, especially those related to epigenetics. These findings have provided a global initiative to disclose the epigenetics mechanisms underlying carcinogenesis and cancer heterogeneity. In contrast to genetic mutations, epigenetics aberrations constitute reversible events which may be restored to their normal state through the application of epigenetic therapy, suggesting the outstanding and promising potential of epigenetic drugs for druggability. Furthermore, the tumor microenvironment also plays a very important role in carcinogenesis since nontumor cells provide a niche that favors the onset of CSC, which lead to heterogeneity, drug resistance, and poor prognosis. Therefore, although anticancer therapies employing classical cytotoxic agents remain in use, epigenetic drugs, which may include substances that modulate REDOX-state, should be considered as an important alternative in monotherapy or even in combination with other drugs.
